# Validation of Doloplus-2 among nonverbal nursing home patients - an evaluation of Doloplus-2 in a clinical setting

**DOI:** 10.1186/1471-2318-10-9

**Published:** 2010-02-20

**Authors:** Karin Torvik, Stein Kaasa, Øyvind Kirkevold, Ingvild Saltvedt, Jacob C Hølen, Peter Fayers, Tone Rustøen

**Affiliations:** 1 Faculty of Nursing, Oslo University College, Oslo, Norway; 2 Department of Cancer Research & Molecular Medicine, Norwegian University of Science and Technology (NTNU), Trondheim, Norway; 3 Faculty of Nursing, Sør Trøndelag University College, Trondheim, Norway; 4 Palliative Medicine Unit, Department of Oncology, St Olavs University Hospital, Trondheim, Norway; 5 Norwegian Centre for Dementia Research, Vestfold Mental Health Care Trust, Tønsberg, Sem, Norway; 6 Faculty of Health and Sports, University of Agder, Kristiansand, Norway; 7 Department of Neuroscience, Faculty of Medicine, University of Science and Technology (NTNU), Trondheim, Norway; 8 Department of Public Health and General Practice, University of Science and Technology (NTNU), Trondheim, Norway; 9 Division of Applied Health Sciences, School of Medicine and Dentistry, University of Aberdeen, Aberdeen, UK; 10 Centre for Shared Decision Making and Nursing Research, Oslo University Hospital, Oslo, Norway

## Abstract

**Background:**

Pain measurement in nonverbal older adults is best based on behavioural observation, e.g. using an observational measurement tool such as Doloplus-2. The purposes of this study were to examine the use of Doloplus-2 in a nonverbal nursing home population, and to evaluate its reliability and validity by comparing registered nurses' estimation of pain with Doloplus-2 scores.

**Method:**

In this cross-sectional study, Doloplus-2 was used to observe the pain behaviour of patients aged above 65 years who were unable to self-report their pain. Nurses also recorded their perceptions of patient pain (yes, no, don't know) before they used Doloplus-2. Data on demographics, medical diagnoses, and prescribed pain treatment were collected from patient records. Daily life functioning was measured and participants were screened using the Mini Mental State Examination.

**Results:**

In total, 77 nursing home patients were included, 75% were women and the mean age was 86 years (SD 6.6, range 68-100). Over 50% were dependent on nursing care to a high or a medium degree, and all were severely cognitively impaired. The percentage of zero scores on Doloplus-2 ranged from 17% (somatic reactions) to 40% (psychosocial reactions). Cronbach's alpha was 0.71 for the total scale. In total, 52% of the patients were judged by nurses to be experiencing pain, compared with 68% when using Doloplus-2 (p = 0.01). For 29% of the sample, nurses were unable to report if the patients were in pain.

**Conclusions:**

In the present study, more patients were categorized as having pain while using Doloplus-2 compared with nurses' estimation of pain without using any tools. The fact that nurses could not report if the patients were in pain in one third of the patients supports the claim that Doloplus-2 is a useful supplement for estimating pain in this population. However, nurses must use their clinical experience in addition to the use of Doloplus-2, as behaviour can have different meaning for different patients. Further research is still needed about the use of Doloplus-2 in patients not able to self-report their pain.

## Background

Pain is a major problem in the nursing home population [1-5]. Previous studies have shown that pain prevalence ranges from 27% to 84% in this population, with the highest rates among patients who are capable of self-reporting pain [2,4,6]. A recent study of Norwegian nursing homes reports pain prevalence as being highest among nonverbal patients [7]. Cognitively impaired patients are reported as receiving fewer analgesics than cognitively intact patients [2,8]. Studies have also shown that neuropathological changes in dementia subtypes may affect the pain experience, where both increased and decreased sensation of pain may occur [9]. In addition, those with severe cognitive impairment may have difficulty in communicating their pain to caregivers, sometimes leading to the mistaken assumption that they are not in pain [1].

Pain is a subjective and persistent phenomenon and the gold standard in pain assessment is to use scales based on the patient's self-report [10]. A clinical definition of pain states that: 'Pain is, whatever the experiencing person says it is, existing whenever he/she says it does' [11]. Several studies suggest that self-reporting scales can be used in dementia populations and that use of these scales can improve pain detection [2,10,12]. However, many of these studies have systematically eliminated patients without the ability to self-report their pain. Given that language loss is inevitable in the most advanced stage of dementia and sometimes following stroke, valid and reliable methods for pain assessment in nonverbal older adults are needed. In these patient populations, other methods, such as behavioural pain observation methods, become more useful and necessary. However, among patients with severe dementia, even behavioural observation methods for capturing pain can be challenging. These patients often have severe physical limitations due to rigidity and contractures that may inhibit them from expressing their pain through behaviours or body language [13].

Pain assessment tools based on behavioural observation methods include observation of changes in behaviour and functioning involving, for instance, sleep, appetite, physical activity, mobility and facial/body language [14,16]. Facial grimaces have been reported by nursing staff as the most important behavioural expression of pain in nonverbal patients [17]. In a study by Cohen-Mansfield and Creedon [17] it was shown that 76% of nursing staff reported that facial grimaces provided the easiest means of detecting pain, while less than half reported signs such as an increase in agitation, moodiness, irritability, pacing or reaction to touching a body part. Furthermore, they relied on knowledge of a resident's habits and needs when differentiating between behaviour attributable to pain and to other factors [17].

A number of studies have focused on the development and validation of tools measuring pain in nonverbal older adults, based on behavioural observation methods. Husebø et al. [18] developed the Mobilization-Observation-Behaviour-Intensity-Dementia Pain Scale (MOBID), a tool for assessing pain behaviour during standardized active, guided movements. The tool rates the presence of pain behaviour indicators, pain intensity, pain localization and overall pain intensity [18]. Measuring pain intensity and localization in addition to pain/no pain will be a huge step forward in pain elucidation in this group. This is also very problematic, because the pain behaviours are not always accurate reflections of pain intensity, and will also indicate other sources of distress, such as physiological or emotional distress [19]. A systematic review in 2006 identified 12 behavioural pain assessment tools [16]. The study reported that the Pain Assessment in Advanced Dementia Scale [20], Pain Assessment Checklist for Seniors with Limited Ability to Communicate [21], Doloplus-2 [22] and L'Échelle Comportementale pour Personnes Âgées [23] showed the best psychometric qualities. However, these tools were still under evaluation and awaiting confirmation of various aspects of their psychometric properties [16]. Herr et al. [24] concluded in a review that Doloplus-2 was a comprehensive tool for assessing pain in nonverbal older adults and that the tool addressed many key indicators noted in the literature and American Geriatrics Society (AGS) guidelines. Hadjistavropoulos et al. [25] developed an interdisciplinary expert consensus statement on assessment of pain in older persons. They maintained that assessment scales were under development and consensus could not be reached regarding the definition recommendation of any particular scale [25].

The Doloplus-2 has been translated into Norwegian and tested in Norwegian nursing home populations [15,26]. The pilot validation study found satisfactory criterion validity when comparing Doloplus-2 with an expert's evaluation of pain [15]. However, the second study by Holen et al. [26] found poor criterion validity in Doloplus-2 when employing a similar design. The authors reported that criterion validity of Doloplus-2 increased when administered by personnel with high levels of competence in pain management in elderly patients with cognitive impairments [26]. However, further validation of Doloplus-2 is needed in populations that cannot self-report their pain.

Therefore, the objectives of the present study were to evaluate Doloplus-2 in a nonverbal nursing home population by:

• examining the use of Doloplus-2 and unscored items, minimum and maximum scores for the tool and subscales;

• evaluating the reliability (internal consistency) of Doloplus-2; and

• comparing registered nurses' estimations of the pain using the Doloplus-2 tool.

## Methods

The data for this study were obtained from a cross-sectional survey designed to explore pain and quality of life in patients in nursing homes in central Norway. The data were collected from September 2005 to May 2006.

In-patients aged 65 years and over from seven Norwegian nursing homes were included in the study. Central Norway consists of three counties (Nord Trøndelag, Sør Trøndelag and Møre and Romsdal). Nursing homes are owned by municipalities and their funding depends on the ranking of priorities in municipal budgets. This can lead to different levels of qualified staff among nursing homes and variations in care, pain assessment and pain management. There are also policy differences among municipalities governing which patients receive nursing-home care rather than community care. To achieve a representative sample, nursing homes were stratified into six strata, by urban and rural status, in each of the three counties in Central Norway. One nursing home was drawn from each stratum. In Møre and Romsdal, two nursing homes were included from rural areas because of low patient numbers in these nursing homes.

Patients were excluded if they had been in the nursing home for less than one week, had a short life expectancy (estimated by the nursing staff) or were younger than 65 years. All eligible patients were scored on Mini Mental State Examination (MMSE) by the investigator (KT), and the patients were categorized as nonverbal if they scored zero on this test.

### Data collection

Demographic data (age, gender, education and marital status), medical diagnoses (including pain-related diagnosis and dementia), and prescribed treatment were collected for all patients from their medical or nursing records by the investigator (KT).

Prescribed medications were classified according to the Anatomical Therapeutic Chemical (ATC) System [27] and were from ATC groups musculoskeletal and analgesic (anti-inflammatory and anti-rheumatic products, topical products for joint and muscular pain, muscle relaxants, anti-gout preparations, drugs for treatment of bone disease, and analgesics). Pain-relieving medication was categorized according to the work done by Nygaard et al. [2]: no opioids, weak opioids, strong opioids, and other pain-related medication (topical products for joint and muscular pain, muscle relaxants, anti-gout preparations, and drugs for the treatment of bone disease).

Pain-related diagnoses were based on previous research and consisted of fractures, knee and hip prostheses, osteoporosis, stroke, cancer, migraine and headache, myalgia, arthritis, amputation, angina pectoris, herpes zoster, low-back pain, duodenal ulcers, ventricular ulcers and gastritis [1,2,28].

### Daily life functioning

Barthel's Activities of Daily Living Index (ADL) was used as a screening instrument for patients' daily life functioning. This 10-point scale measures patients' degree of self-reliance, with a total score ranging from zero to 20. Lower scores indicate greater dependence on nursing care [29]. ADL scores below 5 indicate high dependency, 5 to 8 indicate medium dependency, 9 to 12 indicate low/medium dependency and scores from 13 to 20 indicate low dependency [30]. ADL data were collected from patients' records, by the investigator (KT), and validated by a registered nurse.

### Pain

The Doloplus-2 consists of a list of 10 items divided into three subgroups: five somatic items (somatic complaints, protective body postures adopted at rest, protection of sore areas, expression, sleep patterns), two psychomotor items (washing and/or dressing, mobility) and three psychosocial items (communication, social life, problems of behaviour) [22]. Each item is scored from 0 to 3, where 0 is 'absent' and 3 is 'the highest score of the behaviour'. This gives a range from 0 to 30, with higher scores indicating more pain behaviours. The cut-off score between 'pain' and 'no pain' was set at 5, as recommended by the scale's developers [22]. It is not necessary to have responses for all items on the scale, and the instructions for use of Doloplus-2 emphasize not scoring those items that are judged as inappropriate for a given patient [22].

The Doloplus-2 was administered by the primary nurse (the registered nurse with the best knowledge of the patient's behaviour) [15,22,26]. This nurse cared for the patient regularly and needed to be present for the two days prior to assessing the patient. Before data collection started, the researcher increased staff awareness of patients' pain by teaching about pain and Doloplus-2. Staff received both oral and written information about how to use the Doloplus-2. They were told not to score the items on the Doloplus-2 if they were inappropriate for the given patients and the scoring should only be related to pain behaviours [22]. The scoring should also be done during mobilization, if possible. The researcher was also available to support them during the data collection.

Immediately before applying the Doloplus-2, the primary nurse answered a single question: 'Do you believe that this patient is experiencing pain?' Response options were 'no', 'yes' or 'don't know'. This measurement was done to compare the results with the Doloplus-2 scoring done by the same nurse.

### Ethics

As patients in this study were cognitively impaired, guidelines from the Ministry of Health and Care Service in Norway were followed when recruiting [31]. Relatives of patients or their legal guardians received written information prior to the study and consented on their behalf. Patients received oral information prior to the study and were excluded if they declined to participate, even if their relatives/legal guardians had consented on their behalf.

The Regional Committees for Medical Research in Ethics, Southeast Norway, the Norwegian Social Science Data Services and the Ministry of Health and Care Service approved the study.

### Statistics

Descriptive statistics were used to present demographic and clinical characteristics of the sample and gain an overview of not scored items on Doloplus-2. The percentages of minimum and maximum scores were calculated by assessing the percentages of lowest (0) and highest possible scores on the subscales (6, 9 and 15, respectively) and for the total questionnaire [32]. Mean scores are also calculated for the items, the subscales and the total questionnaire.

Cronbach's alpha coefficients were calculated to assess the internal consistency reliability of Doloplus-2. The alpha coefficient was calculated for the total questionnaire and for subscales. Alpha was also used to explore whether scales could be shortened by the deletion of single items. If Cronbach's alpha changes little when an item is deleted, then the item is a candidate for being removed from the scale [32]. In accordance with Fayers and Machin [30], an alpha above 0.90 was taken to indicate acceptable reliability for individual patient assessment.

The criterion validity was calculated by comparing registered nurses' subjective and Doloplus-2 pain assessments for each patient by Fisher's exact test. Comparison of the groups in respect of pain-related diagnoses and pain management was also calculated by Fisher's exact test.

The statistical software SPSS for Windows v.16.0 was used for analyses. A two-tailed P value below 0.05 was considered statistically significant.

## Results

### Sample

In total, 307 patients were registered in the seven nursing homes. Of these, 41 refused to participate, one patient had been admitted for less than one week, and one was excluded because of short life expectancy. Based on MMSE, 136 nonverbal patients were registered. Of these, 50 did not participate because their relatives/legal guardians refused on their behalf. As Doloplus-2 forms were incomplete for nine patients, 77 patients were included in the present study.

### Demographic and clinical characteristics

The mean age of the sample was 86 years (SD ± 6.6), with a range from 68 to 100 years. The majority of patients were female (75%), 61% were widows or widowers and 20% were married (Table 1). Half the patients had attended only primary school, but education level was unknown for 34% of respondents. Seventy-four per cent had one or more known pain-related diagnoses (Table 1), the most frequent being stroke (31%), fractures (24%), angina (15%), myalgia and arthritis (13%) and cancer (12%). Sixty per cent of the patients received prescribed pain medication (Table 1). Of the 46 patients who received pain medication, 65% received non-opioid analgesics, 11% weak opioids, 13% strong opioids and 11% had other pain medication prescribed, such as topical products for joint and muscular pain, muscle relaxants, anti-gout preparations or drugs for the treatment of bone disease. In addition, 57% (n = 44) received antipsychotics and 39% (n = 30) anti-depressants.

**Table 1:** Demographic and clinical data (n = 77).

		n %
**Gender**		
Male		19 (24.7)
Women		58 (75.3)

**Marital status**		
Not married		9 (11.7)
Married, cohabiting		15 (19.5)
Divorced, separated		3 (3.9)
Widow, widower		47 (61.0)
Missing		3 (3.9)

**Education**		
Primary school		41 (53.2)
More than primary school		10 (13.0)
Missing		26 (33.8)

**Prescribed pain medication**		
No prescribed pain medication		31 (40.3)
No opioid		30 (39.0)
Weak opioid		5 (6.5)
Strong opioid		6 (7.8)
Other pain medication (ACT; M 02, 03, 04, 05)^1^		5 (6.5)

**Pain-related diagnoses**		
Yes		57 (74.0)
No		20 (26.0)

**Barthel index**		
High dependency	(ADL scores <5)	28 (36.4)
Medium dependency	(ADL scores 5-8)	15 (19.5)
Low/medium dependency	(ADL scores 9-12)	20 (26.0)
Low dependency	(ADL scores >12)	13 (16.9)
Missing		1 (1.3)

**Mini Mental State Examination score**		0

^1 ^M02 = topical products for joint and muscular pain, M03 = muscle relaxants, M04 = anti-gout preparations, M05 = drugs for the treatment of bone disease

Scores on the Barthel Index showed that 56% had high or medium levels of dependence on nursing care, about 25% had a low/medium level of dependence, and less than 20% had low dependence (Table 1). None of the patients could complete the MMSE due to severe cognitive impairment (MMSE scores are zero), even though the majority had not been given any dementia diagnosis (Table 1).

### Doloplus-2: unscored items, minimum, maximum and mean score

The number of patients with no scores on the subscales of Doloplus-2 ranged from five (on the subscale psychomotor reactions) to eight on the somatic reactions subscale (Table 2). The percentage of zero scores, indicating absence of the behaviour, ranged from 17% (somatic reactions) to 40% (psychosocial reactions). A maximum score (indicating the highest possible score of behaviour) was found on the psychomotor reactions subscale, but not on other subscales or total Doloplus-2 (Table 2). The mean scores varied from 0.5 to 0.9 for the different items, except for somatic complaints, where the mean score was 1.4. The mean score of the subscales varied from 1.6 on the psychomotor reactions subscale to 3.5 on the somatic reactions scale (Table 2). The mean score for the total scale was 6.9 (Table 2).

**Table 2:** Doloplus 2 score distribution and reliability (n = 77)

	Mean ± SD	Range	Not scored n	Lowest score %	Highest score %	Chronbach's alpha	Alpha if subscale is deleted
**Somatic reactions** 0 indicates absence of behaviour and 15 indicates highest expression of the behaviour	3.5 ± 2.7	0 - 15	8	16.9	0	0.60	

*Somatic complaints*	1.4 ± 1.0						0.47

*Protective body postures adopted at rest*	0.5 ± 0.9						0.53

*Protection of sore areas*	0.5 ± 0.7						0.56

*Expression*	0.7 ± 0.9						0.60

*Sleep pattern*	0.5 ± 0.9						0.56

**Psychomotor reactions** 0 indicates absence of behaviour and 6 indicates highest expression of the behaviour	1.6 ± 1.3	0 - 6	5	24.7	1.3	0.80	

*Washing &/or dressing*	0.9 ± 0.7						0.67

*Mobility*	0.7 ± 0.8						0.67

**Psychosocial reactions** 0 indicates absence of behaviour and 9 indicates highest expression of the behaviour	2.0 ± 2.4	0 - 9	6	40.3	0	0.78	

*Washing &/or dressing*	0.7 ± 1.1						0.72

*Mobility*	0.9 ± 1.1						0.69

*Washing &/or dressing*	0.5 ± 0.8						0.72

**Total scale** 0 indicates absence of behaviour and 30 indicates highest expression of the behaviour	6.9 ± 4.4	0 - 30	0	6.5	0	0.71	

### Reliability (internal consistency)

Cronbach's alpha coefficients were computed for total scale, three subscales and for the subscale when the item was deleted [32] (Table 2). The alpha score for the total questionnaire was 0.71, the psychomotor reactions scale was 0.80, the psychosocial reactions scale 0.78, and the alpha score for the somatic reactions scale was 0.60 (Table 2). When alpha values were calculated after excluding individual items, the alpha values were comparable to alpha for the overall scale, except for the somatic reactions subscale where the alpha score decreased from 0.60 to 0.47 when the somatic complaints item was deleted (Table 2).

### Nursing proxy-rated pain vs. Doloplus-2 proxy-rated pain

In total, 52% (n = 40) of patients were judged by registered nurses to be experiencing pain, and 19.5% (n = 15) as not having pain (Table 3). However, nurses stated that they did not know if the patient was experiencing pain in 29% (n = 22) of the sample. When looking at Doloplus-2 scores, 68% of patients (n = 52) were evaluated as experiencing pain (Table 3). When comparing the two measurements, nursing staff evaluated significantly more patients as experiencing pain when using Doloplus-2 compared with proxy-rated pain (p = 0.01) (Table 3).

**Table 3:** Comparison of the Doloplus-2 score and the Nursing staff rating (n = 77)

Doloplus-2 score	Nursing staff rated pain					
	
	*"No pain"*		*"Pain"*		*"Don't know"*	
	
	(*n = 15*)		(*n = 40*)		(*n = 22*)	
	n	(%)	n	(%)	n	(%)

< 5 ("*no pain*")	11	(73.3)	4	(10.0)	10	(45.5)
	
≥ 5 ("*pain*")	4	(26.7)	36	(90.0)	12	(54.5)

When pain was proxy rated, 36 of 40 (90%) patients in the 'pain' group scored ≥ 5 on Doloplus-2 (indicating pain) and 11 of 15 (73.3%) in the 'no pain' group scored < 5 on Doloplus-2 (indicating no pain). However, more than 50% in the 'don't know' group scored ≥ 5 on Doloplus-2 (indicating pain) (Table 3). In total, 26.7% who scored 'no pain' using proxy rating, scored ≥ 5 (indicating pain) on Doloplus-2, while 97% (n = 36) of the patients who were scored 'in pain' using proxy rating, scored ≥ 5 (indicating pain) on Doloplus-2 (Table 3). In total 70%, 14 out of the 20 patients scoring ≥ 5 (indicating pain) on the psychosocial items was scored 'don't know' with proxy rating.

Neither proxy-rated pain nor Doloplus-2 scores were affected by pain-related diagnosis (proxy-rated pain, p = 0.27 and Doloplus-2, p = 0.79), but significantly more of the 'no pain' group received no prescribed pain medication compared with the 'pain' group (66.7% vs. 32.5%, p = 0.03) in the proxy-rated pain group.

## Discussion

This is one of the first studies evaluating behaviour-based pain tools in a complete nonverbal patient group. The present study included 77 nonverbal nursing home patients, with a mean age of 86 years. The majority of the patients had medium or high level of dependence on nursing care.

Earlier research using Doloplus-2 in cognitive impaired patients concludes that using the scale demands specific administration skills [26]. In the present study, the Doloplus-2 was scored by the primary registered nurse (the nurse with the best knowledge of the patient's behaviour) for each patient. Nurses were trained in using the scale according to the investigator's instructions before they scored the patients, but they lacked experience in using Doloplus-2 in a clinical setting. The best pain estimates should be obtained when using a systematic approach and trained nursing staff who are fully familiar with the patient's behaviour. However, as the Doloplus-2 is observation based it can be difficult to separate specific pain behaviour from other types of behaviour and the pain prevalence may therefore be overestimated with Doloplus-2.

According to the guidelines for Doloplus-2, nursing staff should not score patients on pain if the items are inappropriate or if it is suspected that the patient's change in behaviour is probably not a result of pain, but caused by depression, dependence, or cognitive functioning [22]. We found the highest number of patients not scored occurred on the somatic reactions scale (Table 2). Hølen et al. [15] demonstrated that four of the somatic reactions items (somatic complaints, protective body postures adopted at rest and protection of sore areas) could explain more of the expert variance than the total Doloplus-2. The fact that patients in the present study were more cognitively impaired than the sample in the study by Hølen and colleagues [15] could explain some of the differences in the results. In the present study, the highest congruence between the Doloplus-2 score and the proxy rating score was found on the psychomotor score as nine out of ten patients scored as 'pain' by proxy rating were scored five or more on Doloplus-2 (indicating pain). This result suggests that nurses are good at interpreting psychomotor reactions as pain indicators in persons with severe dementia, in spite of these patients being partly or totally immobile and in high dependence on nursing (Table 1). It could also indicate that the nurses overestimated the patients' pain by incorrectly interpreting psychomotor behaviours as pain indicators.

We observed the highest percentage of minimum scores on the psychosocial reaction scale (31 out of 71) and the maximum score was on the psychomotor reactions scale (Table 2). Hølen et al. [15] demonstrated that the three psychosocial items did not add substantially to the total pain score. In the present study, the highest percentage of minimum scores (indicating no pain) was found on this item. We found the highest congruence between Doloplus-2 score >5 (indicating pain) and the proxy rating score 'don't know' on the psychosocial scale. This could indicate that psychosocial reactions in persons with severe dementia are not very specific for pain, because psychiatric and behavioural symptoms are highly prevalent among these patients [33], and therefore not good pain indicators.

When estimating internal consistency reliability of the Doloplus-2 in the present study, Cronbach's alpha coefficients were between 0.47 and 0.72, the lowest being for the somatic reactions subscale. These values are all below the alpha scores recommended by Fayers and Machin [32] for individual patient assessment, and below the alpha coefficient recommended for the total Doloplus-2 questionnaire [22], which is above 0.80. Another study estimating internal consistency in 501 older adults from centres participating in the Doloplus Group [22] found a Cronbach's alpha of 0.82 for the total scale. Chen et al. [34] found a Cronbach's alpha between 0.67 (psychosocial reaction subscale) and 0.87 (psychomotor reactions subscale) on the different subscale. The Cronbach's alpha for the total scale was 0.74 [34].

The items in Doloplus-2 are clearly heterogeneous, reflecting a variety of important issues or dimensions. It could therefore be problematic to achieve a high Cronbach's alpha coefficient in a nonverbal population. In the present study, the alpha scores obtained after deleting an item were comparable with the overall alpha coefficient for the scales, except for somatic complaints (Table 2), implying that each of the items contributes similarly to the construct it is intended to measure [32]. The somatic complaints items contribute more to the construct somatic reactions than do protective body postures adopted at rest, protection of sore areas, expression and sleep pattern.

A high proportion of the patients were estimated to have pain: between 52% and 68% depending on which measure was used. Consequently, the nursing staff evaluated significantly more patients as having pain when using Doloplus-2 compared with proxy-rated pain in this nursing home population. Thus, one can speculate that Doloplus-2 is a more sensitive measure than proxy rating. Another explanation for that the prevalence of pain being 42% higher when using Doloplus-2, could be that the nurses were forced to make a decision about patients' pain experience even when uncertain, because the Doloplus-2 does not provide a 'don't know' option in their scale.

Estimating pain in this population is challenging and might lead to limitations in the present study. The patients in the present sample had reduced cognitive function, reduced verbal capacity, changes in pain experience due to dementia and received medication that could influence their behaviour (e.g. antipsychotics and antidepressants). In addition, 56% had high or medium dependence on nursing care. All these factors can influence patients' behaviour and thus impact on the observations made of them. Only a minority of the patients had a dementia diagnosis in their records, but all were subjectively judged as 'patients with dementia' by the nursing staff. This is in concordance with the study of Selbæk et al. [33] that showed that only half of the patients with dementia in Norwegian nursing homes had a dementia diagnosis in their record. In the same study, it was demonstrated that 72% of patients with dementia had clinically significant delusions, hallucinations, anxiety, aggression/agitation, apathy, disinhibition, irritability or aberrant motor behaviour. It is therefore a challenge to separate the specific behaviours caused by pain in this population. More research is needed on the interaction between pain symptoms and these behaviour problems [33]. To evaluate the patient's pain behaviour, it is essential to know patients' normal behaviours. Every patient has his/her 'pain signature', and this signature may change as the dementia progresses. Even if this area is challenged by many contributing variables, it is of great importance to continue the research as there is evidence that this group is in great pain. One strength in the present study was that the nurses who did the scoring knew the patients.

In our study, neither proxy rating nor Doloplus-2 were affected by pain-related diagnosis, but significantly more of the 'no pain' group received no pain medication compared with 'pain' group in the proxy rated. Previous studies have demonstrated that proxy-rating assessment can be affected by knowledge of whether patients were receiving prescribed pain medication [2]. This might result in patients being wrongly categorized as not having pain and not receiving pain medication even if they need it. In a study including 125 patients from three Norwegian nursing homes, the nurses reported pain in 39% of the nonverbal patients [2]. The administration of pain medication was significantly (p = 0.001) associated with reporting 'pain' [2], which indicated that nurses' pain ratings can be affected by knowledge of prescribed pain medication. However, the explanation of the congruence between prescribed pain medication and 'pain' could also be a result of the staff being very good judges of patients' pain behaviours. The patients who needed pain medications received prescribed pain medications, but in insufficient dosis. Husebø [18] concluded that patients with severe dementia received less pain relief than they needed and that they had higher prevalence of ICD diagnosis than patients with mild and moderate dementia. Nevertheless, more research is needed about this connection.

One limitation in this study was the relatively small sample size. With a larger sample, the threshold level of five could also have been evaluated. There are no published studies evaluating the Doloplus-2 using a cut-off point of five for separating patients with or without pain. However, pain cannot be ruled out if a patient is a few points below the cut-off point scores, because patients may have different 'pain signatures' [22]. It is a limitation that the developers based their cut-off point between 'pain' and 'no pain' solely on clinical experience [22].

One may question the use of nonverbal patients for evaluating Doloplus-2 in the present study. Previous studies have included both communicative and nonverbal patients provided they had reduced communicative function [15,26]. Doloplus-2 is a nonverbal assessment tool based on behavioural observation methods, with focus on changes from patients' normal behaviour. In the present study, the tool was tested in a sample of nonverbal patients who in addition showed changes in their behaviour due to dementia and reduced cognitive function. This is in accordance with the clinical setting, and reflects the clinical challenge health-care workers face. One strength of the present study is the use of different approaches when validating Doloplus-2. Proxy rating of pain (by patient's primary nurse), Doloplus-2 rating of pain (by the same nurse), registration of diagnoses related to pain and prescribed pain medication are included in the validation process.

### Clinical implications

Concordance (90%) was found between proxy rating and Doloplus-2 scores with respect to estimating 'pain' with the two different assessment methods, suggesting that the two measures are addressing the same pain construct. About half of the patients for whom nurses rated their pain prevalence as 'don't know' when using proxy rating were scored ≥ 5 (indicating pain), and less than half scored < 5 (indicating no pain) with Doloplus-2. The influence of prescribed pain medication was reduced when staff used Doloplus-2 instead of proxy rating. Previous research has demonstrated that the prevalence of identified pain decreases with increasing cognitive function, despite similar prevalence of pain-related diagnosis [1,8]. The designer of Doloplus-2 emphasizes that, if in doubt, one should not hesitate to conduct a test treatment with an appropriate analgesic [22]. Even if it is accepted that a score ≥ 5 is a sign of pain, for border-line scores the patient should be given the benefit of the doubt. If the patient's behaviour changes following analgesic administration, pain is indeed involved.

In the present study, more patients were categorized as having pain using Doloplus-2 compared with nurses' estimation of pain without any tool. Previous studies have demonstrated that proxy rating of pain without using an assessment scale leads to underestimating of patients' pain [1,2]. The fact that nurses could not report whether the patients were in pain or not in one third of the patients strongly supports the use of Doloplus-2 as a supplement to proxy rating. However, further research is still needed about the use of Doloplus-2 in those patients who are not able to self-report their pain.

## Conclusion

Based on the results from this and previous Norwegian studies, we recommend the use of Doloplus-2 in addition to proxy rating when assessing pain in patients without the ability to self-report. There may be a risk of overestimating patients' pain with Doloplus-2 and a risk of underestimating pain when using only proxy assessment. However, it is better to overestimate rather than underestimate pain and when in doubt, behaviour changes following analgesic administration may resolve the question of whether pain is indeed present. It appeared that nursing staff's pain estimation was influenced by their knowledge of prescribed pain medication, and there was a risk of under-reporting pain when using the proxy assessment on patients not currently receiving pain medication. Therefore, behavioural observation methods should be used as only one strategy to help inform nurses about pain in older nonverbal persons. Overall, further research is needed on pain in the elderly, and especially in nonverbal patients, to further improving their pain management.

## Competing interests

The authors declare that they have no competing interests.

## Authors' contributions

KT, SK, ØK and TR contributed to the design of this study. KT and TR organized and performed the data collection. KT, PF and TR performed the statistical analysis. All authors participated in interpretation of the data, drafting the manuscript and all read and approved the final manuscript.

## Pre-publication history

The pre-publication history for this paper can be accessed here:

http://www.biomedcentral.com/1471-2318/10/9/prepub
